# Augmentation of cardiac sympathetic tone by percutaneous low-level stellate ganglion stimulation in humans: a feasibility study

**DOI:** 10.14814/phy2.12328

**Published:** 2015-03-24

**Authors:** Olujimi A Ajijola, Kimberly Howard-Quijano, Jennifer Scovotti, Marmar Vaseghi, Christine Lee, Aman Mahajan, Kalyanam Shivkumar

**Affiliations:** 1 UCLA Cardiac Arrhythmia CenterLos Angeles, California; 2 Neurocardiology Research Center of ExcellenceLos Angeles, California; 3 Department of Anesthesia, University of California-Los AngelesLos Angeles, California

**Keywords:** Activation recovery interval, arrhythmias, stellate ganglion, sympathetic stimulation

## Abstract

Modulation of human cardiac mechanical and electrophysiologic function by direct stellate ganglion stimulation has not been performed. Our aim was to assess the effect of low-level left stellate ganglion (LSG) stimulation (SGS) on arrhythmias, hemodynamic, and cardiac electrophysiological indices. Patients undergoing ablation procedures for arrhythmias were recruited for SGS. A stimulating electrode was placed next to the LSG under fluoroscopy and ultrasound imaging; and SGS (5–10 Hz, 10–20 mA) was performed. We measured hemodynamic, intracardiac and ECG parameters, and activation recovery intervals (ARIs) (surrogate for action potential duration) from a duodecapolar catheter in the right ventricular outflow tract. Five patients underwent SGS (3 males, 45 ± 20 years). Stimulating catheter placement was successful, and without complication in all patients. SGS did not change heart rate, but increased mean arterial blood pressure (78 ± 3 mmHg to 98 ± 5 mmHg, *P* < 0.001) and dP/dt max (1148 ± 244 mmHg/sec to 1645 ± 493 mmHg/sec, *P* = 0.03). SGS shortened mean ARI from 304 ± 23 msec to 283 ± 17 msec (*P* < 0.001), although one patient required parasympathetic blockade. Dispersion of repolarization (DOR) increased in four patients and decreased in one, consistent with animal models. QT interval, T-wave duration and amplitude at baseline and with SGS were 415 ± 15 msec versus 399 ± 15 msec (*P* < 0.001); 201 ± 12 msec versus 230 ± 28 msec; and 0.2 ± 0.09 mV versus 0.22 ± 0.08 mV, respectively. At the level of SGS performed, no increase in arrhythmias was seen. Percutaneous low-level SGS shortens ARI in the RVOT, and increases blood pressure and LV contractility. These observations demonstrate feasibility of percutaneous SGS in humans.

## Introduction

The autonomic nervous system powerfully regulates all aspects of cardiac function, and its electrophysiology (Vaseghi and Shivkumar 2008). Excitation of the sympathetic nervous system (SNS) has been linked to atrial and ventricular arrhythmogenesis (Schwartz 2014; Shen and Zipes 2014). In animal models, direct electrical and chemical stimulation of neural elements within the cardiac sympathetic nervous system, most commonly the stellate ganglia, have provided significant insights into the possible mechanisms by which sympathoexcitation-mediated arrhythmias originate in humans (Yanowitz et al. 1966; Meyer et al. 2010; Ramirez et al. 2011; Ajijola et al. 2013b). Pharmacologic and reflex sympathetic stimulation (Vaseghi et al. 2012), as well as anesthetic blockade of the stellate ganglia have also been used to modulate ventricular electrophysiology and to treat arrhythmias (Schwartz et al. 1976; Cinca et al. 1985; Fujii et al. 2004; Tan et al. 2012), however, studies on direct stimulation of the sympathetic nervous system in humans are lacking.

High-frequency electrical stimulation in the proximal pulmonary artery in humans induces premature ventricular contractions (PVCs) from the right ventricular outflow tract in the presence of dobutamine (Hasdemir et al. 2009). Mental stress has also been shown to shorten action potential duration (APD). Prolongation of the QT interval was also observed following application of radiofrequency energy to the stellate ganglia during surgery in patients with palmar hyperhidrosis (Wong 1997). Percutaneous stimulation of stellate ganglia (SGS) in humans and the assessment of myocardial electrophysiologic parameters however, have not been performed. In this study, we report the feasibility of percutaneous low-level SGS for studying the modulation of cardiac function and electrophysiology by sympathoexcitation in humans.

## Methods

### Patient population

The study population included five patients referred for cardiac electrophysiologic procedures. The study was approved by the UCLA Institutional Review Board (IRB). All patients received the consent form for the study to review >1 week before presenting for the procedure and written informed consent was signed by all patients. Detailed clinical history, examination, and echocardiographic assessment were performed. Two of five patients were on antiarrhythmic medications; oral Metoprolol Tartrate 25 mg twice daily (held for 1 week prior) in one patient; and carvedilol 12.5 mg twice daily in another patient. None of the patients had a history of autonomic disorders, and had not previously undergone any neuromodulation procedures.

### Electrophysiologic study

Monitored Anesthesia Care (MAC) was provided to all study patients using intravenous propofol infusions (50–100 mcg/kg/min), titrated to moderate sedation. Supplemental oxygen via facemask was delivered to all patients. Electrophysiologic catheters were placed via percutaneous vascular access (via right internal jugular or femoral venous access). An arterial sheath (4F) was placed in the femoral artery. All patients had continuous 12-lead electrocardiographic recordings. A decapolar catheter was placed in the coronary sinus, while quadripolar catheters were placed in the high right atrial, right ventricular, and His bundle positions under fluoroscopic guidance. From the His bundle catheter, atrioventricular conduction [Atrial to His signals (AH interval)] and His-Purkinje conduction [His signal to earliest electrocardiographic QRS complex (HV interval)] were measured. A duodecapolar catheter (20-pole) was placed in the right ventricular outflow tract.

Stellate ganglion stimulation was performed prior to the electrophysiology study and catheter ablation.

### Catheter placement and stellate ganglion stimulation

Once the patients were under MAC, a stimulating peripheral nerve catheter for left SGS was inserted via Tuohy needle as recently described Abdi et al. (2004). In the supine position, the patient's head was extended, and rotated slightly to the right. The skin was cleaned with chlorhexidine prior to application of sterile drapes. Using anterior-posterior fluoroscopy, the C5-6 disk was well visualized, then the left C6 and C7 were identified. Using a linear ultrasound probe (Narouze et al. 2007), the trachea, thyroid, carotid, and internal jugular vein were identified at the C6 level. Lidocaine (1%) was injected to anesthetize skin. With the bevel directed caudal and lateral, an 8 cm, insulated Tuohy-tip needle (Stimucath, Arrow International) was inserted through the lidocaine skin wheel. Using ultrasound guidance, the needle tip was advance so that the needle tip was anterior to the longus colli muscle and avoiding vascular structures and adjacent to T1. After negative aspiration, 1 mL of contrast was injected and checked under fluoroscopy to show adequate spread without vascular entry. 19G StimuCath catheter was then placed through the Tuohy needle. Placement of the StimuCath catheter was confirmed with fluoroscopy between the left C7-T1 levels. The Tuohy needle was removed carefully out of the skin and the catheter was secured with Tegaderm dressing. A nerve-stimulating clip was attached to the proximal end of the nerve-stimulating catheter and attached to an external pulse generator for stimulation (Model #5388, Medtronic, Minneapolis, MN).

Stimulation was initiated at 5 Hz, 5 mA and titrated until a hemodynamic response was observed (increase in blood pressure), typically >20–30 sec of stimulation. Stimulation parameters for each subject are shown in the table.

From the arterial line, hemodynamic monitoring was performed, and a >10% increase in mean arterial pressure was used as a marker for SGS. From the arterial pressure waveform, the rate of rise in pressure over time (dP/dt) was taken as a surrogate for contractility, as it was not measured directly with conductance catheter.

### Data acquisition and activation recovery interval analysis

Unipolar electrograms were recorded from the duodecapolar catheter placed in the right ventricular outflow tract using an electrophysiologic acquisiton system (Cardiolab Electrophysiologic Recording System, General Electric, Fairfield, CT). Recordings were filtered at 0.05–500 Hz. Activation recovery interval (ARI) analysis was performed as previously described Ajijola et al. (2013b).

### Statistical analysis

Values are expressed as mean ± standard error. ARIs before and during SGS in the same subject were analyzed with a Wilcoxon signed-rank test. Comparisons with more than two groups were performed with the Welch's ANOVA, given the non-normal distribution. The values across subjects were compared using the one-tailed Wilcoxon signed-rank test. A *P* value ≤0.05 was considered statistically significant.

## Results

### Patient characteristics

**Figure 1:**
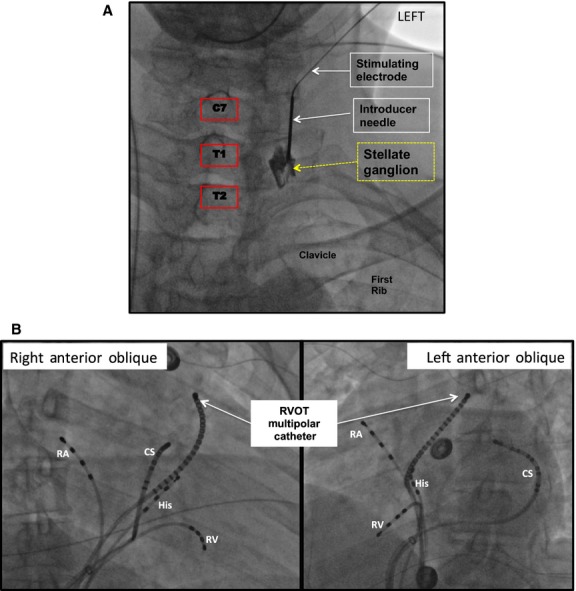
Stimulation and Recording Electrode Set Up. (A) Antero-posterior (AP) fluoroscopic view of the lower cervical and upper thoracic region, depicting the approach and positioning of the introducer needle and StimuCath electrode for stellate ganglion stimulation. Prior to stimulation the introducer needle was removed and the stimulating electrode left in place. (B) Right and left anterior oblique fluoroscopic projections of the thorax, displaying the location of the high right atrial (RA), His bundle, right ventricular (RV), coronary sinus (CS), and right ventricular outflow tract catheters (RVOT). Unipolar electrograms were recorded with the RVOT catheter.

Five patients referred for electrophysiology study and ablation underwent SGS (age 45 ± 20 years; three males). The characteristics of the study patients are shown in the table. All patients had structurally normal hearts, mean left ventricular ejection fraction 62 ± 3.6%. Percutaneous placement of the SGS Catheter was successful in all patients (Fig.1A) and no complications occurred. Electrophysiologic catheters were placed as demonstrated in Fig.1B. Long-term follow up of all patients (16–28 months poststimulation) has shown no late complication following SGS.

### Hemodynamic and intracardiac conduction responses to SGS

**Figure 2:**
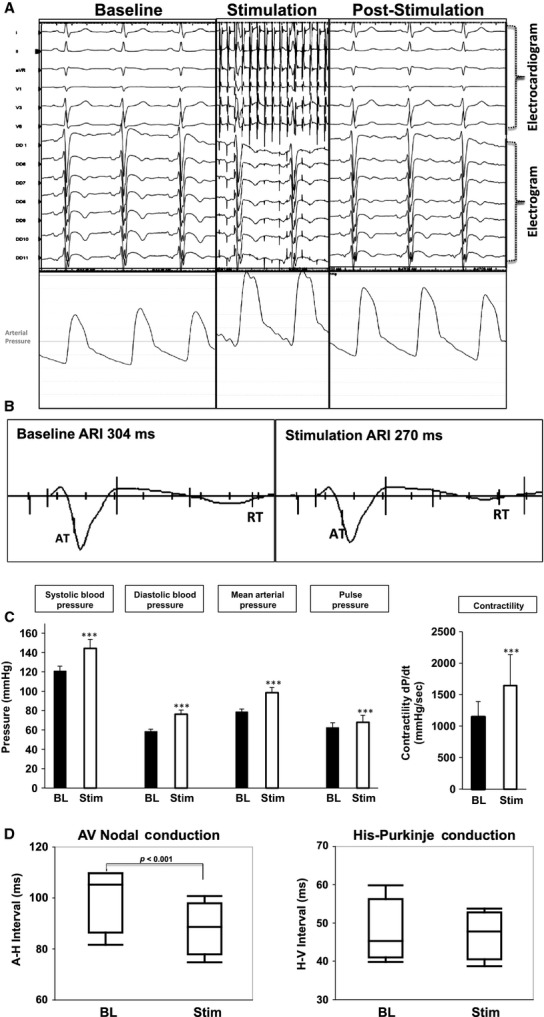
Hemodynamic and Electrophysiologic Response to Sympathetic Stimulation. (A) Representative electrocardiographic, electrogram, and hemodynamic responses to left stellate ganglion stimulation (SGS). (B) An example of ARI measurement from electrogram at baseline and following SGS. The bars on the QRS downslope and T-wave upslope reflect the interval measured as the ARI. (C) Quantifications of Systolic, diastolic, mean arterial, and pulse pressures at baseline and during sympathetic stimulation. dP/dt, a marker for cardiac contractility is also shown. ***P <* *0.001. (D) Graphical quantification of atrio-ventricular nodal (A–H: atrio-His) and His-Purkinje (H–V: His-ventricle) conduction at baseline and following SGS (msec: milliseconds).

Stimulation was initiated at 5 Hz and 5 mA for 20–60 sec, and was confirmed by the appearance of stimulation artifact saturating the electrocardiographic and intracardiac electrogram channels (Fig.2A). The frequency and output were increased gradually after a brief poststimulation reequilibration period (1–5 min), until a hemodynamic response occurred (≥10% increase in mean arterial pressure) (Fig.2A). Stimulation parameters at which this hemodynamic response occurred are listed in Table1. The intention was low-level SGS, therefore, stimulation parameters were not increased further.

**Table 1:** Study subject demographics and stimulation parameters

Subject	Age/Gender	LV EF	Reason for EP study	Stimulation parameters
1	35/F	60%	PVC Ablation	10 mA, 5 Hz
2	19/M	63%	SVT Ablation	20 mA, 5 Hz
3	37/F	70%	PVC Ablation	20 mA, 10 Hz
4	71/M	69%	PVC Ablation	10 mA, 10 Hz
5	59/M	50%	PVC Ablation	20 mA, 5 Hz

SGS increased systolic blood pressure (120.8 ± 5.3 mmHg vs. 144.4 ± 9.2 mmHg, *P *<* *0.001); diastolic blood pressure (58.4 ± 2.6 mmHg vs. 76.4 ± 4.3 mmHg, *P *<* *0.001); mean arterial pressure (78.8 ± 2.8 mmHg vs. 98.6 ± 5.4 mmHg, *P *<* *0.001); and pulse pressure (62.4 ± 5.3 mmHg vs. 68 ± 7.2 mmHg, *P *<* *0.001) (Fig.2C). SGS also increased dP/dt_max_ (a surrogate for cardiac contractility) from 1148 ± 244 mmHg/sec to 1645 ± 493 mmHg/sec (*P *=* *0.03).

SGS did not significantly affect heart rate (70.8 ± 5 beats/min vs. 73.3 ± 6 beats/min, *P *=* *0.15) but shortened the AH interval (100.8 ± 6.6 msec vs. 88.5 ± 5.3 msec, *P *<* *0.001), and did not significantly affect the HV interval (47.8 ± 4.3 msec vs. 47.3 ± 3.2 msec, *P *=* *0.2) (Fig.2D).

### SGS induces ARI shortening

**Figure 3:**
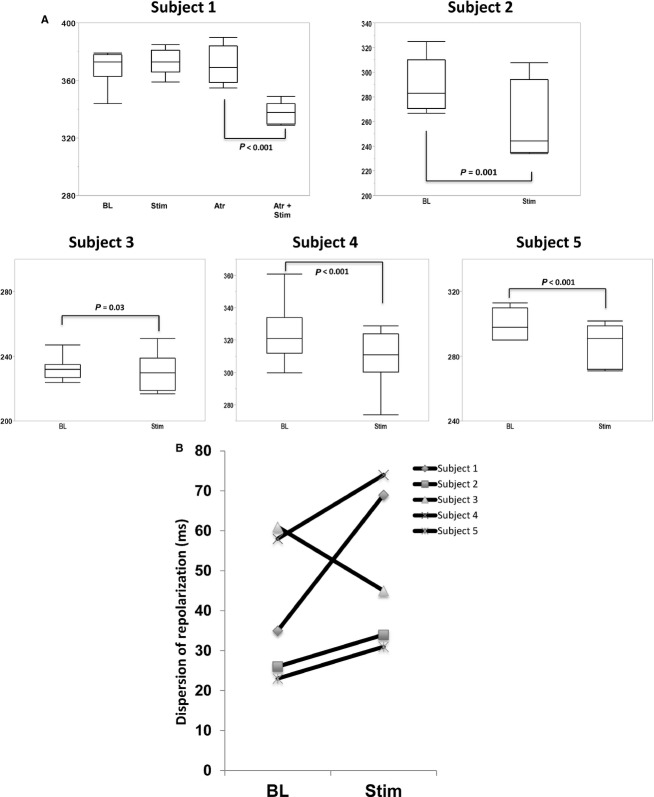
Changes in Activation Recovery Intervals with Stellate Ganglion Stimulation. (A) Individual responses to sympathetic stimulation, with and without atropine (atr) at baseline and during stellate ganglion stimulation. (B) Dispersion of repolarization (ARI_max_–ARI_min_) in each subject.

A representative example of ARI shortening with SGS is shown in Fig.2B. Mean ARI at baseline across all subjects was 303.7 ± 22.5 msec. Individual responses to SGS are shown in Fig.3A. Mean ARI shortened in all subjects except subject one, in whom a trend toward an increase in ARI was observed. This subject also had the highest ARI at baseline (370 msec vs. 230–320 msec for the other subjects). To exclude the potential of parasympathetic influence, atropine 1 mg was administered. Atropine resulted in a slight increase in ARI (364 ± 6 msec vs. 371 ± 12 msec, *P *=* *0.03) (Fig.3C). SGS, in the presence of atropine, resulted in a significant shortening in ARI (371 ± 12 msec vs. 330 ± 20.9 msec, *P *<* *0.001). In all the other subjects, SGS resulted in ARI shortening (Fig.3A), without need for atropine. Dispersion of ARI across all electrodes, taken as the difference between the maximum and minimum ARI values was increased in all patients except one (Fig.3B).

### Influence of SGS on electrocardiographic parameters

**Figure 4:**
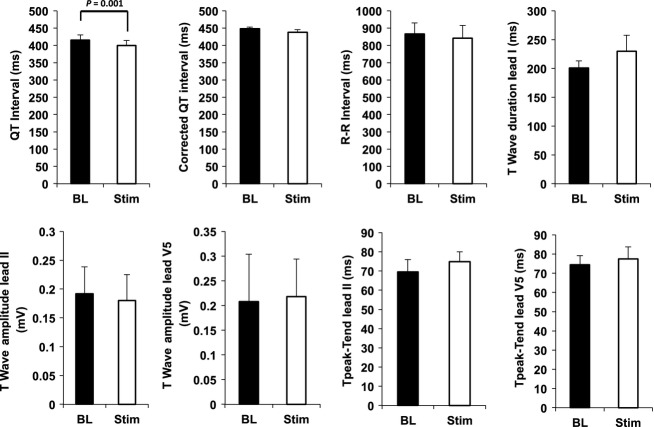
Electrocardiographic parameters during stellate ganglion stimulation. Shown are the mean responses across all subjects to stellate ganglion stimulation in QT interval, corrected QT interval (QTc); R-R interval, T-wave duration, T-wave amplitude in Leads II and V5, and Tpeak-Tend in Leads II and V5 (msec: milliseconds; mV: millivolts).

From the 12-lead electrocardiogram recorded by the Cardiolab system, the QT and corrected QT (QTc) intervals; RR interval; T-wave duration and amplitude; and T_peak_-T_end_ duration were measured. SGS shortened QT interval (515.3 ± 15.1 msec vs. 399.6 ± 15 msec, *P *<* *0.001) but did not significantly change QTc (447.9 ± 5 msec vs. 437.9 ± 7.3 msec, *P *>* *0.2); R-R interval (865.3 ± 64.3 msec vs. 841.3 ± 72.9 msec, *P *>* *0.2); T-wave duration (201 ± 12 msec vs. 229.8 ± 27.6 msec, *P *>* *0.2); or T-wave amplitude (0.2 ± 0.09 mV vs. 0.22 ± 0.08 mV, *P *>* *0.2) (Fig.4).

## Discussion

The main findings of the present study are as follows: (1) low-level SGS using a percutaneous ultrasound- and fluoroscopy-guided stimulating catheter is feasible, does not result in severe arrhythmias, and can be safely achieved without complications; low-level left SGS results in (2) inotropic but not chronotropic response; (3) predominantly an increase in repolarization heterogeneity; and (4) shortening of RVOT ARI and QT interval, but not QTc, T-wave amplitude or duration. These findings demonstrate that percutaneous SGS is a useful approach for studying the influence of sympathoexcitation in humans. To our knowledge, this work represents the first demonstration of percutaneous direct stellate ganglion stimulation in humans.

### Placement of stimulation catheter

Anesthetic blockade of the stellate ganglion has long been used for treating a variety of clinical conditions including complex regional pain syndromes and ventricular arrhythmias (Ackerman and Zhang 2006; Hayase et al. 2013). Percutaneous stellate ganglion access can be achieved safely, although it is critical to avoid the vertebral artery. We adapted this commonly used technique, and slightly modified it by advancing the introducer needle slightly more caudal. For the infusion of an anesthetic, the needle does not need to be advanced quite to the level of the first thoracic vertebra, however, to achieve adequate proximity for stimulation, this is necessary. As noted, no complications occurred with adapting the method described by Abdi et al. (2004).

### Hemodynamic responses to left SGS

Our findings are in line with studies from animal models in that electrical stimulation of the left stellate ganglion (LSG) increases inotropy but not chronotropy, while right SGS results in both chronotropy and inotropy (Yanowitz et al. 1966; Winter et al. 2012; Ajijola et al. 2013b; Zhou et al. 2013). Ueda et al. (1964), in a canine model observed chronotropic responses to both right and left SGS, although it was greater with right than left SGS. Interestingly, Wong (Wong 1997) observed the opposite findings, where left SGS resulted in inotropy and chronotrophy, and right SGS induced inotropy only. This discrepancy may be related to the mechanism of stimulation (5 Watt electrocautery) or the patient population (hyperhidrosis), in whom dysautonomia may be present. The inotropic findings in the present study are consistent with the expected effects of left SGS seen in multiple studies in animal models (Ramirez et al. 2011; Winter et al. 2012; Ajijola et al. 2013b; Zhou et al. 2013).

### Electrophysiologic responses to sympathetic stimulation

In response to SGS, we observed shortening of the QT interval. In a porcine model of sympathoexcitation, similar observations were made, where QT (specifically, ST interval) shortens, with no significant impact on the QTc (Ajijola et al. 2011; Ramirez et al. 2011). In canines, Yanowitz et al. observed QT interval prolongation following left stellate ganglionectomy. Interestingly, they also observed QT prolongation during left SGS. In a noninvasive model of sympathoexcitation in humans, Findler et al. (2010) also observed QT interval shortening. Wong observed shortening of the QTc, although the QT itself is not reported (Wong 1997). These discrepant findings are explained by the study of Abildskov, where both prolongation and shortening of the QT interval were observed (Abildskov 1976). Prolongation was observed when sympathetic stimulation was performed for short durations 3–5 sec, and shortening induced by prolonged stimulations 30 sec to 5 min. Stimulation was performed for over 20–30 sec in our study, as the marker for sympathoexcitation for enhanced mean arterial pressure. In the Wong study, stimulation was performed for 2 sec (Wong 1997). The mechanism of QT shortening induced by SGS, likely relates to overall global shortening in action potential duration (Terrenoire et al. 2005; Ajijola et al. 2013a; Yagishita et al. 2015) and/or enhanced inotropy (Huang et al. 1992, 1995), as we did not observe an increase in heart rate.

The influence of autonomic tone on intracardiac conduction has long been recognized, and stimulation of parasympathetic elements within the human heart, has been used to modulate AV conduction (Bianchi et al. 2009). In this study, despite unchanged heart rates, we observed approximately 10% shortening in the AH interval with no demonstrable effect on the HV interval. This finding would be expected, given the extensive innervation of the AV node (Crick et al. 1994; Chow et al. 2001), and already rapid conduction in the Purkinje fibers. At the level of sympathetic stimulation achieved in the present study, an effect on AV nodal but not Purkinje conduction may be expected.

ARI shortening during SGS has been shown in animal models (Yoshioka et al. 2000; Ajijola et al. 2013a), and in response to emotional, reflex, and direct sympathetic activation in humans (Selvaraj et al. 2009; Vaseghi et al. 2012; Child et al. 2014). In our study, we demonstrated ARI shortening to percutaneous SGS recorded in the RVOT. Functional studies in porcine and canines demonstrate that left cardiac sympathetic innervation predominates on the posterior, inferior, and apical aspects of the ventricles, while the right predominates on the anterior, superior, and base of the ventricles (Yanowitz et al. 1966; Ramirez et al. 2011; Ajijola et al. 2013b) both on the epicardium and endocardium (Yagishita et al. 2015). Although panoramic mapping of the human ventricles to SGS has not been performed, extrapolating from these animal studies, RSG stimulation may have yielded a greater increase in ARI shortening measured in the RVOT. It is also likely that ARIs measured in the posterior aspect of the ventricles may also have shortened more than the RVOT. The degree of ARI shortening seen with left SGS in our study is consistent with prior studies in porcine subjects (Ajijola et al. 2013b; Vaseghi et al. 2013), where approximately 20–40 msec of ARI shortening occurred in the RVOT epicardium with left SGS, by greater than that observed by Child et al. (2014) who noted a +7 to −15 msec change in ARI. This difference likely relates to greater sympathoexcitation by low-level stimulation in our study, relative to mental challenge in the study by Child and colleagues.

Spatial dispersion of repolarization (DOR) is thought to be a marker for increased risk of ventricular arrhythmias (Han and Moe 1964; Kuo et al. 1983). In the present study, DOR was defined as the difference between the maximal and minimal ARI recorded in the RVOT. Left SGS was associated with increased DOR in four out of five subjects, with the fifth subject showing a decrease. In canine and porcine studies (Opthof et al. 1991; Ajijola et al. 2013a), DOR in response to SGS across animals exhibited similar variability, with some animals showing an increase, and others, a decrease. Another marker of dispersion heterogeneity, Tpeak-Tend (Tp-Te), was measured. Tp-Te in the limb leads is thought to reflect global DOR, while Tp-Te in the precordial leads reflects transmural DOR (Antzelevitch et al. 2007). We, therefore, assessed Tp-Te in both limb and precordial leads, but found no statistically significant increase.

The implications of the present study are multiple. First, the majority of our understanding of human cardiac autonomic regulation come from extrapolations of animal studies, or from studies of indirect (reflex) or mental stress-induced sympathoexcitation in humans. That percutaneous direct stellate ganglion stimulation can be achieved safely without significant complications from catheter placement, or from life-threatening arrhythmias or hemodynamic disturbances, suggests that this method of sympathoexcitation may be a valuable research tool. This is especially true since four of the subjects presented for ablation of symptomatic and frequent premature ventricular contractions. Second, hemodynamic, electrocardiographic, and electrophysiologic measures of sympathetic stimulation could be readily obtained and interpreted, indicating that the set up utilized in this study can be applied relatively readily in other settings. Additionally, validation of a variety of assumptions and extrapolations on the workings of the cardiac SNS can be performed by directly stimulating the stellate ganglion without the need for surgical exposure of the ganglia.

## Limitations

The present study is limited by the small sample size, although appropriate for the demonstration of feasibility. Electrograms were recorded from the RVOT only, therefore, the impact of SGS on ARIs in other regions of the heart was not examined. Additionally, SGS was performed using a unipolar set up, resulting in saturation of the surface and intracardiac recordings. This limited the assessment of dynamic changes during SGS.

## Conclusions

In summary, the findings of the present study indicated that percutaneous low-level SGS in humans is feasible, has no acute or long-term complications, and yields meaningful hemodynamic, electrocardiographic, and electrophysiologic data. One patient was on carvedilol (last dose was the day prior to procedure), however, a significant response to SGS was still observed in BP, dP/dt, and ARI. Further studies utilizing direct stellate ganglion stimulation are warranted to validate existing animal data, and to identify novel physiologic functions of the sympathetic nervous system in man.

## Conflict of Interest

None declared.
